# Endoscopic Lavage of Extensive Chronic Subdural Hematoma in an Infant After Abusive Head Trauma: Adaptation of a Technique From Ventricular Neuroendoscopy

**DOI:** 10.7759/cureus.2258

**Published:** 2018-03-02

**Authors:** Thomas Beez, Ann Kristin Schmitz, Hans-Jakob Steiger, Christopher Munoz-Bendix

**Affiliations:** 1 Department of Neurosurgery, Medical Faculty, Heinrich-Heine-University

**Keywords:** pediatric neurosurgery, neuroendoscopy, subdural hematoma, abusive head trauma

## Abstract

Subdural fluid collections are frequently encountered in young children after non-accidental injury. In a subset of patients, these collections progress in size and ultimately require permanent drainage, which is commonly achieved with subdural-peritoneal shunts. However, excessive protein and cellular contents in the fluid are potential risk factors for shunt failure. Here, we describe the adaptation of an endoscopic lavage technique established for ventricular endoscopy with the aim of improving fluid condition prior to shunting. We present a case of subdural fluid collections secondary to non-accidental injury, where permanent shunting was required but could not be performed due to excessive protein and cellular levels in the subdural fluid despite conventional burr hole drainage. A two-month-old male infant presented with a bulging and tense fontanel, a reduced level of consciousness, bradycardia, and significant macrocephaly. Computed tomography (CT) demonstrated massive bilateral, low attenuation subdural fluid collections, reaching a diameter of 4.5 cm. Emergency burr hole washout and insertion of subdural drains was performed. Despite prolonged drainage over 10 days, the protein level remained at 544 mg/dl and the mean erythrocyte count at 6,493/µl. Continuous drainage was required to avoid clinical deterioration due to raised intracranial pressure; however, the fluid condition was still considered incompatible with permanent subdural-peritoneal shunting. We, therefore, performed an endoscopic subdural lavage with a careful evacuation of residual blood deposits. No complications were encountered. Postoperatively, mean protein level was 292 mg/dl and mean erythrocyte count was 101/µl. Endoscopic lavage could be safely performed in a case of extensive subdural low attenuation fluid collections, where conventional burr hole drainage failed to improve protein and cellular contents as a prerequisite for successful permanent shunting. We conclude that adaptation of this technique can be helpful in selected cases as an alternative procedure.

## Introduction

Subdural fluid collections are frequent findings in children after non-accidental traumatic head injury, including acute subdural hematoma, chronic subdural hematoma, low attenuation subdural collections, and intermediate or overlapping stages suggestive of repeated significant head injuries [1-2]. In cases of space-occupying acute subdural hematoma, neurosurgical interventions include craniotomy or craniectomy [3]. Burr hole drainage with or without drain insertion, as well as transcutaneous subdural taps, are frequent treatment modalities in subacute or chronic subdural hematomas, depending on patient age [3]. However, in a subset of patients, subdural collections progress despite intermittent evacuation and ultimately require permanent fluid diversion. This is usually achieved by subdural shunt placement [1, 3]. However, as combinations of clotted subdural blood and increasing low attenuation subdural fluid can occur, the fluid levels of protein and red blood cells (RBC) can be significantly elevated compared to normal cerebrospinal fluid (CSF) [4]. While moderately elevated CSF protein and RBC counts do not appear to increase the risk of shunt failure, excessively high levels or presence of particles and clotted blood presumably pose a risk of catheter or valve occlusion [5].

A comparable scenario is encountered in neonates with intraventricular hemorrhage and subsequent post-hemorrhagic hydrocephalus. Blood and its degradation products are considered the causative agents of post-hemorrhagic hydrocephalus and might additionally contribute to the increased risk of shunt failure in this population [6]. Among several approaches to reduce the proportion of shunt-dependent children and positively influence the risk of shunt failure, neuroendoscopic intraventricular lavage appears to be a promising neurosurgical approach [7]. This technique allows for direct visualization of hematoma removal and irrigation, as well as for active hemostasis of active bleedings. Additionally, the endoscope can be directed towards accumulations of blood degradation products and free-floating particles for lavage or blood clots which can be aspirated. Neuroendoscopic lavage has been shown to be a safe and feasible procedure with a positive influence on shunt rate and reduction in subsequent surgical interventions [7].

We describe the adaptation of neuroendoscopic lavage for the treatment of a progressive hemorrhagic subdural fluid collection in an infant with a non-accidental head injury.

## Technical report

**Figure 1:**
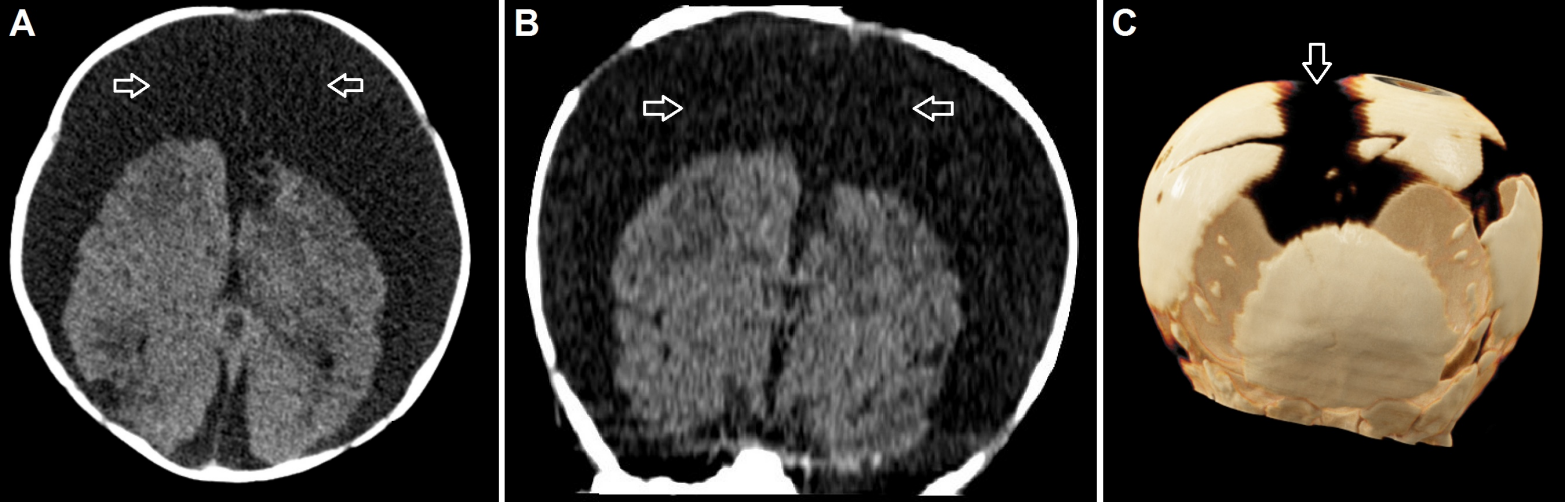
Preoperative cranial imaging Preoperative cranial computed tomography scans demonstrating excessive bilateral subdural low attenuation fluid collections (arrows) in the axial (A) and coronal (B) planes, as well as a three dimensional reconstruction (C) showing macrocephaly with split cranial sutures (arrow).

A two-month-old male infant presented with a bulging and tense fontanel, split cranial sutures, a reduced level of consciousness, bradycardia, and significant macrocephaly. Computed tomography demonstrated massive bilateral low attenuation subdural fluid collections, reaching a diameter of 4.5 cm, with focal hyperdense areas corresponding to subacute blood clots (Figure 1). Emergency burr hole washout and insertion of bilateral subdural drains was performed. However, despite prolonged external subdural drainage over 10 days, the protein levels remained between 506 - 600 mg/dl (normal value: 20 - 50 mg/dl) and RBC counts between 3,555 - 9,356/µl (normal value: < 5/µl). The child required continuous external drainage because clamping of the drains repeatedly resulted in clinical deterioration due to raised intracranial pressure. The fluid condition was considered incompatible with permanent subdural-peritoneal shunting, not only because of the unfavourable protein and RBC levels but also due to the macroscopically visible particles and blood clots which were noted in the drained fluid or had even occluded the external drains, necessitating sterile flushing. We, therefore, adapted the established neuroendoscopic ventricular lavage technique published by Schulz et al. and performed a neuroendoscopic subdural lavage, aiming at the improvement of fluid conditions as a prerequisite for successful subdural shunting [7].

**Figure 2:**
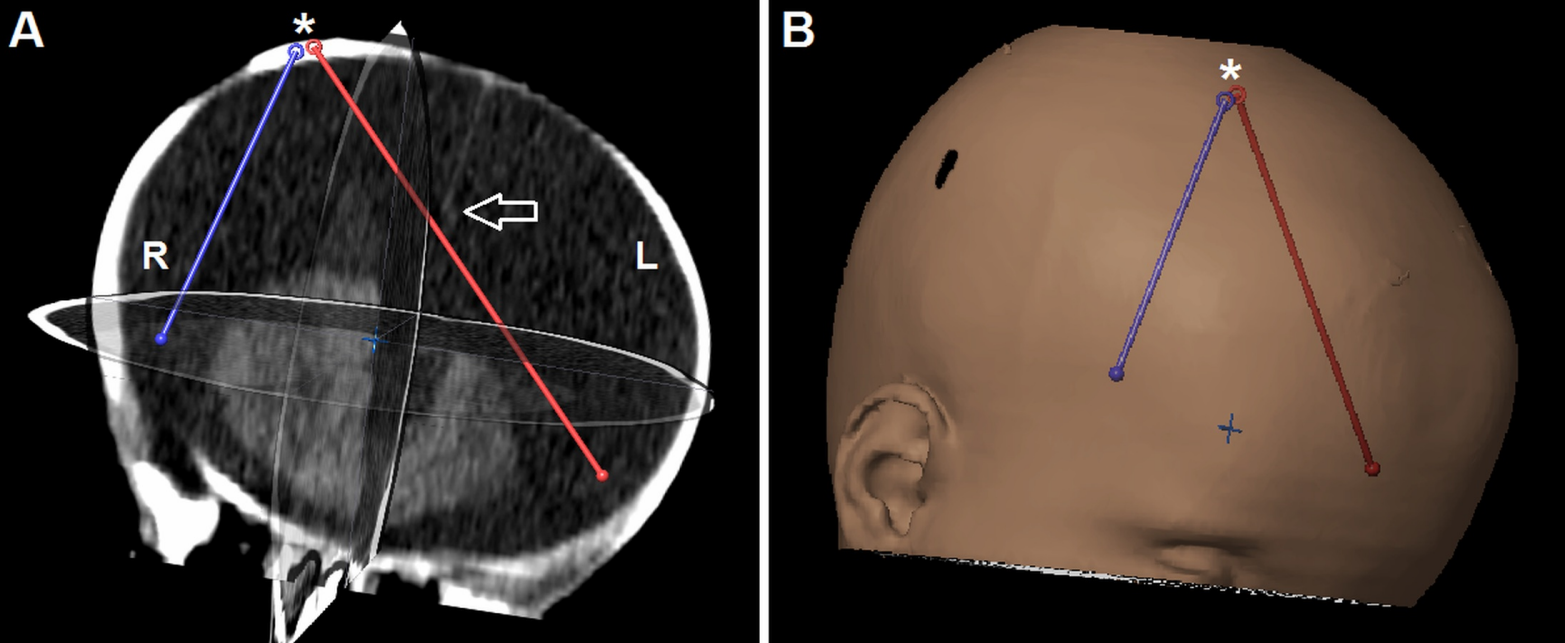
Preoperative virtual planning Preoperative virtual planning based on cranial computed tomography (A), showing the entry point (*) and endoscopic trajectories (red and blue lines). Of note, the red trajectory allows safe passage of the endocope below the falx (arrow) to reach the contralateral subdural space. (B) A three dimensional reconstruction demonstrates the right frontal coronal entry point.

**Figure 3:**
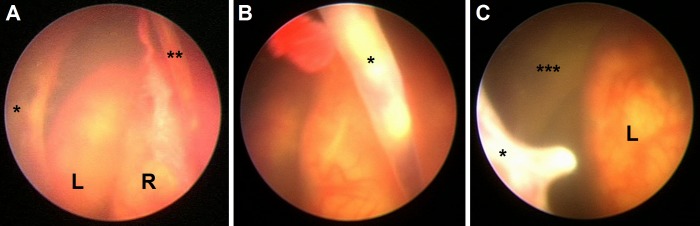
Intraoperative neuroendoscopic views Endoscopic views obtained with a 0° rigid endoscope inserted through a right coronal burr hole: (A) Both cerebral hemispheres (L/R), the falx (*), and a stretched bridging vein (**) are visualized; (B) close-up image of a stretched bridging vein (*), revealing thrombosis; (C) once the endoscope is passed between the falx (*) and the left hemisphere (L), the contralateral middle cranial fossa (***) can be accessed. L: left; R: right

Preoperative virtual planning of the procedure was performed using iPlan® Net 3.0.0 neuronavigation software (BrainLAB AG, München, Germany) to accurately determine the optimal entry point and trajectories (Figure 2). After induction of general anaesthesia, the child was positioned supine with the head fixed in a head ring. Prophylactic single shot intravenous antibiotics were administered (cefuroxime at 50 mg/kg). After sterile preparation and draping, the right frontal burr hole originally made for conventional hematoma evacuation was re-opened and used as an entry point for the Minop® 0° rigid endoscope (B Braun Aesculap AG, Tuttlingen, Germany). The endoscope was advanced into the massively dilated subdural compartment, and the cerebral hemispheres, falx, and stretched and thrombosed bridging veins were visualized under continuous irrigation with a passive flow of sterile lactate-free Ringer's solution warmed to 37°C (Figure 3). Passive outflow through an empty working channel assured balanced intracranial volume without an increase in intracranial pressure. In addition to passive lavage, the endoscope was directed towards blood deposits and clots, which were actively aspirated by attaching a syringe to the endoscope’s working channel. Due to the significant macrocephaly with massively dilated subdural space, the contralateral side was accessed by advancing the endoscope under the falx with great caution to avoid injury to bridging veins (Figure 3). After irrigation with approximately 3,000 ml of Ringer solution, no accessible blood deposits were visible and the overall visibility improved significantly. No immediate complications, such as active bleeding, were encountered. After retraction of the endoscope, the cortical tract was closed with a gelatine sponge, the dura was reconstructed using Tachosil® (Takeda Pharmaceutical Co. Ltd, Osaka, Japan), and a standard two-layer wound closure was performed. Postoperatively, subdural fluid samples were collected via the remaining contralateral external drain, with protein levels now ranging between 244 - 320 mg/dl and erythrocyte counts between 29 - 154/µl. Therefore, subdural shunting was then considered feasible and a Bactiseal® catheter system (DePuy Synthes, Raynham, MA) with an adjustable proGAV® 2.0 valve (Christoph Miethke GmbH & Co. KG, Potsdam, Germany) were implanted. The postoperative course was unremarkable, and no evidence of shunt occlusion or infection was present within the first 30 days following shunting. A stable clinical improvement of the signs of active hydrocephalus with raised intracranial pressure was achieved and the clinical findings, including the status of the fontanel and cranial sutures, as well as head circumference, improved.

## Discussion

Neuroendoscopic lavage of the subdural space was, in this case, a feasible, safe, and effective approach to reduce protein level, RBC count, and macroscopic debris from the subdural fluid after non-accidental head injury. We, therefore, conclude that this technique offers an effective approach to the rare combination of progressive subdural collections not deemed suitable for permanent shunting.

The impact of elevated CSF protein and RBC levels on shunt failure rates remains controversial, with some studies negating a significant association both in children with post-hemorrhagic hydrocephalus, as well as in adults with hydrocephalus after aneurysmal subarachnoid hemorrhage [8-9]. In contrast, several authors recommend delaying permanent shunting for post-hemorrhagic hydrocephalus until the CSF protein content is below 150 - 200 mg/dl [6, 10]. In the present case, macroscopic debris and blood clots were visible in the subdural fluid, in addition to very high RBC and protein levels. We, therefore, estimated the risk of shunt occlusion to be increased, justifying an alternative approach.

While acute subdural hematoma is the most common pathological finding in children after non-accidental head injury, persistent or progressive low attenuation fluid collections develop only in a subgroup of patients. Of these, approximately 40% ultimately require permanent fluid diversion [2]. The scenario encountered in this case report is uncommon with regard to the excessive extent of head circumference enlargement and diameter of the subdural collection. However, treatment in such extreme cases can be challenging. In our experience, the combination of conventional burr hole drainage, escalation to neuroendoscopic lavage, and finally, implantation of a subdural shunt with programmable differential pressure valve with the gravitational unit was successful in achieving a durable clinical improvement and control of the progressive head growth. We hope that by further valve adjustments in the future excessive macrocephaly can be prevented, which would interfere with head control, motor development, reaching milestones, such as free seating, and also social acceptance and might ultimately require surgical salvage techniques, such as reduction cranioplasty [11].

A prerequisite for neuroendoscopic subdural lavage is certainly a significant enlargement of the subdural space, allowing for insertion and movement of the endoscope. Additionally, a unilateral approach is only possible if the distance between brain surface and falx or dura is wide. Otherwise, a bilateral endoscopic approach should be considered, placing burr holes in a fashion that allows safe access to the relevant clots or blood deposits, which, in our case, were located along the middle and anterior fossa. Angled rigid endoscopes might be helpful in addition to a standard 0° endoscope. An alternative to rigid neuroendoscopy could be the use of flexible endoscopes, which have been used before to fenestrate subdural membranes in adult septated chronic subdural hematomas [12]. Caution is required to avoid injury to the bridging veins, which are severely stretched in such cases. The bridging veins play an important role in abusive head trauma, as they are susceptible to acceleration/deceleration, rotational, and shear forces and their subsequent rupture causes subdural hemorrhage [13]. Of note, bridging vein thrombosis has also been described as a sequel to head injury and was observed in the present case.

## Conclusions

Neuroendoscopic lavage could be safely performed in a case of massive subdural low attenuation fluid collections with blood deposits, where conventional burr hole drainage failed to improve protein and cellular contents as a prerequisite for successful permanent shunting. We conclude that adaptation of this technique can be helpful in selected cases as an alternative procedure.
